# A Unique Pathogen Causing Infective Endocarditis in a Three-year-old Girl

**DOI:** 10.7759/cureus.4249

**Published:** 2019-03-13

**Authors:** Apoorva Ravindranath Waikar, Omosede Uzamere, Kaneisha Bailey, Louisdon Pierre, Adebayo Adeyinka

**Affiliations:** 1 Pediatrics, The Brooklyn Hospital Center, Brooklyn, USA; 2 Pediatrics, The Brookyln Hospital Center, Brooklyn, USA

**Keywords:** infective endocarditis, invasive pneumococcal disease, pediatric infections, pneumococcus, pneumococcal endocarditis, pediatric infective endocarditis, pediatric cardiology

## Abstract

The overall incidence of infective endocarditis (IE) in adults has been reported to be 1.5 to 6.0 per 100,000 patient-years. In children, the incidence of IE in the general population is approximately three times lower. The presence of cyanotic congenital heart disease is considered to be the most strongly associated risk factor to develop IE. In approximately 8% to 10% of pediatric cases, IE develops without structural heart disease or any other readily identifiable risk factors. In these situations, the infection usually involves the aortic or mitral valve secondary to *Staphylococcus aureus* bacteremia. *Streptococcus pneumoniae* endocarditis in a female with no known risk factors is extremely rare and has no established optimal therapy. We hereby present a case of a three-year-old girl, with no identifiable risk factors diagnosed with IE caused by *S. pneumoniae*.

## Introduction

Infective endocarditis (IE) occurs less commonly in children than in adults. A study by Pasquali et al. noted the annual incidence rate for IE in children in the United States to be between 0.05 and 0.12 cases per 1000 pediatric admissions from 2003 to 2010 [1]. The epidemiology of heart disease in children has changed during the past three to four decades. Rosenthal et al. in a retrospective study over a 12-year period noted that the proportion of subjects infected with Streptococcal species has declined, whereas the proportion of those infected with Staphylococcal species increased in the post-antibiotic era [2]. Because of the increased survival rate of children with congenital heart disease and the overall decrease in rheumatic valvular heart disease in developed countries, congenital heart disease now constitutes the predominant underlying condition for IE in children over the age of two years in developed countries [3].

## Case presentation

A three-year-old female presented to our emergency department with a three-week history of productive cough, rhinorrhea, non-bloody non-bilious emesis, and intermittent fevers with a maximum temperature of 102 °F. The child was treated for pneumonia on two occasions in the past six months. There was also a history of recurrent ear infections and she was diagnosed to have moderate persistent asthma a year ago. Her birth history was uncomplicated and there was no history of any structural heart disease or any other congenital defects. The child was up to date with vaccines including four doses of pneumococcal conjugate vaccine (PCV)13. There was no family history of recurrent infections, immunodeficiency, consanguinity, or cardiac problems.

On detailed physical exam, subcostal retractions were noted with crackles and decreased air entry on the right side. In light of the respiratory distress, a chest radiograph was obtained and demonstrated an opacification in the right lower lobe, and hence she was admitted with a diagnosis of pneumonia. The following morning, a new soft 2/6 systolic murmur was auscultated over the cardiac apex. Cardiac echocardiography was performed, which revealed 3-mm vegetation on the anterior mitral valve leaflet. Initial laboratory evaluation showed a white count of 23 X10^3^/cu.mm^3^, with 83.4% neutrophils. Her C-reactive protein (CRP) and erythrocyte sedimentation rate (ESR) were 131.17 mg/dL and 35 mm/hr, respectively. Human immunodeficiency virus (HIV) test was resulted to be negative. The primary blood culture grew *Streptococcus pneumoniae*, serotype 3N sensitive to penicillin G and ceftriaxone. Based on the clinical finding of a new murmur with cardiac vegetations on the echocardiogram and the blood culture report, the patient was diagnosed to have IE and was treated with IV ceftriaxone for four weeks. CBC and CRP were trended throughout her hospital stay until her leukocytosis resolved and CRP normalized. Follow-up echocardiogram one month later showed resolution of vegetation and the murmur.

Final diagnosis 

The final diagnosis was IE due to *S. pneumoniae*, serotype 3N.

## Discussion

IE in the absence of coronary heart disease (CHD) is often associated with central indwelling venous catheters. In addition to CHD, the high-risk groups for IE in pediatric population include children with noncardiac congenital malformations, malignancies, genetic syndromes, and prematurity and those who have been treated by either invasive procedures including intravenous medications [3-5]. In approximately 8% to 10% of pediatric cases, IE develops without structural heart disease or any other risk factors and usually involves the aortic or mitral valve secondary to Staphylococcus aureus bacteremia [6]. The incidence of IE in children with no risk factors is showing an upward trend although the exact numbers are unclear [5]. A Canadian study by Marrie et al. on 3,251 adult patients with invasive pneumococcal disease (IPD) showed the rate of pneumococcal endocarditis complicating IPD to be 0.86% [7]. Endocarditis due to *S. pneumoniae* accounts for 3% to 7% of all cases of childhood endocarditis [2].

Researchers from the Mayo Clinic studied the characteristics of IE in 97 children from 1950-2011. *Staph. aureus* and *Viridans streptococci* were the most common pathogens isolated [8]. Only one out of these 97 had pneumococcal endocarditis; however, the presence or absence of risk factors in this subject is unknown. This finding is backed by researchers, Lin et al., and a systematic review by Vogku et al., who note that apart from Viridans Group *Streptococci* and *Staph. aureus*, coagulase-negative *Staphylococci* (CoNS) is the third most common pathogen of IE [9-10]. A five-year study in Japan by Ishiwada et al. comprising of 170 children with endocarditis had two boys with no risk factors with a pneumococcal etiology [11]. Givner et al. published the findings of US Pediatric Multicenter Pneumococcal Surveillance Group who prospectively identified children seen at eight major centers with invasive disease due to *S. pneumoniae* during the period of 1st September 1993 through 28th February 2003 [12]. During the 10-year period, the study group identified a total of 3,065 children with pneumococcal bacteremia of which only 11 cases of pneumococcal endocarditis (0.4%) were identified. Of these 11 cases, only one child had no history of structural heart disease [12]. In concordance with other studies and adult data, a male preponderance was noted with 73% of the subjects being males [12-13]. Our patient, being a female with no history of any structural heart defect or other risk factors, makes this an unusual presentation of pneumococcal endocarditis.

Community pathogens, such as *S. aureus*, *S. pneumoniae*, *Kingella kingae*, and *Hemophilus* species have been found to affect children with no predisposing risk factors [5]. Three studies review IE in children with no structural heart disease and predisposing factors [5,9,14]. The characteristics of endocarditis vary in children with and without risk factors. The most striking finding is the acute fulminant course and a high complication rate in children with no predisposing factors [15]. Although considered an uncommon infection, pneumococcal endocarditis has been associated with high mortality rates [16-17]. High mortality rate might be present despite rapid eradication of the pathogen from the blood with complications manifested by severe CHF and embolic phenomena, mainly to the central nervous system (CNS) secondary to large left-sided vegetations [5,18].

The management of IE in children comprises both medical as well as surgical interventions. While parenteral antibiotic therapy is still the first-line treatment, due to the rapid progression of pneumococcal IE, early and timed surgical intervention may be essential to improve survival [19-20]. The use of surgery is necessary in case of cardiac complications such as heart failure, perforation, rupture or abscess of the valve, endocarditis on prosthetic valve, or embolic complications. Other indications for surgical interventions include fungal endocarditis, certain gram-negative bacilli (*Serratia marcescens*, *Pseudomonas* spp.), or in cases of persistent bacteremia despite good antibiotic therapy [20].

## Conclusions

Pneumococcal endocarditis should be considered among children with no known risk factors for IE. Because of the potential for multidrug resistance and the infrequency of pneumococcal IE, no optimal therapy has been established for this illness. Treatment is usually cephalosporin alone or a combination of third- or fourth-generation cephalosporin with vancomycin. Early isolation of the pathogen with antibiotic sensitivity and IV antibiotics provides a greater chance of resolution of the disease process. This appears consistent with the findings in the scientific statement from the American Heart Association.
